# Disease Ecology of Rickettsial Species: A Data Science Approach

**DOI:** 10.3390/tropicalmed5020064

**Published:** 2020-04-27

**Authors:** Serge Morand, Kittipong Chaisiri, Anamika Kritiyakan, Rawadee Kumlert

**Affiliations:** 1 CNRS ISEM—CIRAD ASTRE—Montpellier University, 34090 Montpellier, France; 2 Faculty of Veterinary Technology, Kasetsart University, Bangkok 10900, Thailand; anamika.k@ku.ac.th; 3 Department of Helminthology, Faculty of Tropical Medicine, Mahidol University, Bangkok 10400, Thailand; kittipong.cha@mahidol.ac.th; 4 The Office of Disease Prevention and Control 12, Songkhla Province (ODPC12), Department of Disease Control, Ministry of Public Health, Songkhla 90000, Thailand; rawadee.k@ddc.mail.go.th

**Keywords:** *Rickettsia*, *Orientia*, *Ehrlichia*, *Anaplasma*, rickettsial diseases, ticks, mammals, scrub typhus, disease ecology, network analysis, data science, EID2

## Abstract

We present an approach to assess the disease ecology of rickettsial species by investigating open databases and by using data science methodologies. First, we explored the epidemiological trend and changes of human rickettsial disease epidemics over the years and compared this trend with knowledge on emerging rickettsial diseases given by published reviews. Second, we investigated the global diversity of rickettsial species recorded in humans, domestic animals and wild mammals, using the Enhanced Infectious Disease Database (EID2) and employing a network analysis approach to represent and quantify transmission ecology of rickettsial species among their carriers, arthropod vectors or mammal reservoirs and humans. Our results confirmed previous studies that emphasized the increasing incidence in rickettsial diseases at the onset of 1970. Using the Global Infectious Diseases and Epidemiology Online Network (GIDEON) database, it was even possible to date the start of this increase of global outbreaks in rickettsial diseases in 1971. Network analysis showed the importance of domestic animals and peridomestic mammals in sharing rickettsial diseases with humans and other wild animals, acting as important hubs or connectors for rickettsial transmission.

## 1. Introduction

Rickettsioses are infectious diseases caused by the obligate intracellular Gram-negative bacteria, mainly transmitted through bites of infected arthropod vectors such as ticks, fleas, lice and mites [1,2]. Rickettsial diseases include anaplasmosis, ehrlichiosis, spotted fever, typhus fever, scrub typhus and the understudied trematode-borne neorickettsiasis [3]. Several comprehensive reviews have been published in the recent years on rickettsial diseases [4,5], spotted fever [6], tick-borne rickettsioses [7,8] and scrub typhus [9,10,11,12].

Studies have emphasized that the incidence of tick-borne rickettsial diseases has increased over the last 40 years with at least for four endemic rickettsioses: Rocky Mountain spotted fever, Mediterranean spotted fever, North Asian tick typhus, and Queensland tick typhus (QTT). These rickettsial diseases have shown a marked, continuous increase in incidence since 1970 [13]; the increase in incidence of scrub typhus has been reported to have started more recently [14].

Parola et al. [7] summarized current knowledge on tick-borne rickettsioses, caused by obligate intracellular bacteria belonging to the spotted fever group of the genus *Rickettsia,* following a geographic approach and incorporating background information on history, epidemiology and diagnostics given from a previous review [15]. The methodology was to extract information in publication databases (PubMed) with search terms of word combinations such as “ticks”, “rickettsia”, “rickettsioses”, “spotted fever” and “typhus”, a common methodology used in review articles.

Here, we presented a different approach to assess the disease ecology of rickettsial species by investigating open databases and by using data science methodologies.

First, we explored the dynamics of worldwide epidemics of rickettsial species, using the GIDEON database (Global Infectious Disease and Epidemiology Online Network, www.gideononline.com), which has been used in several recent comparative studies [16,17]. Our main aims using this database were to explore the epidemiological trend and changes of rickettsial disease epidemics over the years and to compare this trend with knowledge on emerging rickettsial diseases given by the review of Jones et al. [18] and more recently by Swei et al. [19].

Second, we investigated the diversity of rickettsial species using a second database, the Enhanced Infectious Disease Database (EID2) [20] (https://eid2.liverpool.ac.uk). The purpose was to use a network analysis approach, which has already been shown to be of interest in representing and quantifying transmission ecology of pathogens among different individuals or different host species [21]. Network architectures of pathogens and their carriers, along with associated indices, were used here to investigate rickettsial species, their vectors and reservoirs and their transmission to humans. Modularity in bipartite and unipartite networks of pathogens in vectors and in reservoirs, respectively, that share common pathogen species may help to assess the potential risks of pathogen transmission to humans [22,23], and network centrality indices can provide useful information on the relative importance of a given element in a network to the structure of the whole system [23]. A given carrier (reservoir or vector) occupying a highly central position (i.e., high centrality value) in a given network may behave like a hub or a connector by linking different carriers clustered into subgroups within the network. Finally, identifying carriers with high values of centrality in networks may help in targeting key vectors or reservoirs for rickettsial disease surveillance [23].

## 2. Materials and Methods

### 2.1. Data Acquisition

To explore the epidemics of rickettsial species, we used the GIDEON database. The list of outbreaks of microbial and parasitic diseases per country was extracted from this database, which contains information on the presence and occurrence of epidemics for each country. This dataset has been regularly used in previous comparative studies of pathogen diversity and epidemics [16,17]. We used the package ‘segmented’ [24] implemented in the freeware R [25] for detecting a breakpoint in the trend of outbreaks of human rickettsial diseases from 1920 to 2016.

To explore the emerging rickettsial species, we used the datasets of emerging infectious disease events compiled by Jones et al. [18] between 1940 and 2004 and Swei et al. [19] between 1920 and 2016.

To explore the diversity of rickettsial species, we used the EID2 (see Supplementary Materials). The datasets were extracted from the database, using species interactions and species distribution, based on studies published between 1950–2012. The quality of the EID2 database was verified by comparing with other datasets of pathogens infecting arthropods, humans, domestic animals and wild mammals (for details, see [20]).

### 2.2. Analysis

We applied network-based methodologies, which have previously been widely used in epidemiology; disease ecology; and pathogen transmission across human, wildlife or livestock populations [21,26]. We estimated the hosts which are potential sources of rickettsial agents by investigating the network topology of shared rickettsial agents among carriers (reservoirs/vectors), vertebrates or arthropods. We used degree centrality, which is defined as the number of secondary links to a node in a network, corresponding to the number of ties that a given host has with other hosts. Degree centrality can be interpreted in terms of the immediate risk of a host being infected by a rickettsial agent circulating through the network. A central host (i.e., with high value of centrality) is the one that is infected by many rickettsial species that also infect or are shared with many other hosts in the network.

We used bipartite networks where nodes describing hosts interact with nodes describing pathogens. We projected these bipartite networks onto unipartite networks using the ‘tnet’ package [27] implemented in R. A unipartite network represents patterns of relative interactions amongst carriers through the sharing of rickettsial species. Each host within a network plays a different role in rickettsial species sharing relative to all other nodes in the network. The role of each host within the network was examined using its centrality measurement. A central node (a carrier) is the one that is highly connected to other nodes (carriers) and thus is supposed to have a greater transmission potential for rickettsial species. A carrier with high centrality means that this carrier is highly connected to other carriers and thus is likely to have a greater potential of rickettsial transmission to several other carriers. We calculated the eigenvalue centrality (EC) with the ‘evcent’ function from the igraph package [28] in R.

We built two bipartite networks (presence/absence of a link) linking carriers: (1) mammal species, including humans, with all rickettsial species and (2) arthropod vectors with all rickettsial species. We then transformed these bipartite networks where separate nodes from hosts or vectors were connected with nodes of rickettsial species to unipartite networks using the tnet package in R [25]. We used the function ‘cluster_louvain’ implemented in the package igraph [28] to identify the modularity structure of the unipartite networks. This function is based on a multilevel modularity optimization algorithm [29]. We also extracted the path lengths among hosts (nodes) for each network.

We tested the phylogenetic signal in the centrality values for shared rickettsial species among carriers (reservoirs and vectors) with phylogenetic information obtained using ‘rotl’ [30] retrieved from the Tree of Life [31], phylogenetic trees manipulated using ‘ape’ [32], ‘phytools‘ [33], phylobase’ [34] and phylogenetic signal obtained with ‘phylosignal’ [35]; all packages were implemented in R.

## 3. Results

### 3.1. Outbreaks of Human Rickettsial Diseases

The GIDEON database recorded the information on epidemics for the following rickettsial diseases: anaplasmosis, African tick bite fever, human monocytic ehrlichiosis, Japanese spotted fever, rickettsialpox, New World spotted fever, Old World spotted fevers, endemic typhus, epidemic typhus and scrub typhus.

**Figure 1:**
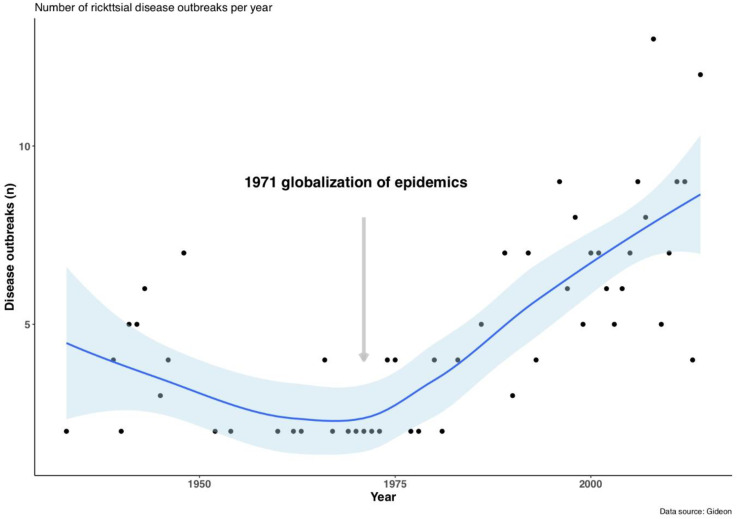
Trends in outbreaks of rickettsial diseases per year from 1920 to 2016, with significant increase in the number of outbreaks since 1971 (using the package ‘segmented’ [24]). Data on rickettsial disease outbreaks were obtained from the GIDEON database (see Section 2).

The number of worldwide outbreaks recorded by year showed an increasing trend from 1920 to 2016 (Figure 1). The start of this change was dated in 1971, with less than five outbreaks per year in average from 1920 to 1970 and with outbreaks reaching around 10 per year around 2015.

### 3.2. Emerging Rickettsial Diseases

The lists of emerging infectious diseases gathered by Jones et al. [18] and Swei et al. [19] indicated several rickettsial agents that have emerged from 1920 to 2016 (Table 1). Some likely causes of emergence were demography, war and famines. Causes of greater importance include global travels and trade and land use changes, with few mentions of the effects of human susceptibility to infection or climate change (Table 1).

### 3.3. Rickettsial Diversity in Space and Among Reservoirs, Vectors and Humans

**Figure 2:**
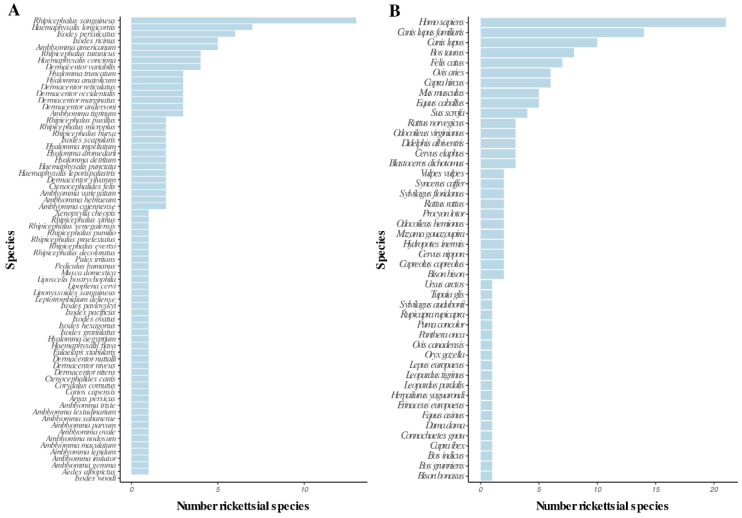
Number of rickettsial species by carriers: (**A**) arthropod species; (**B**) mammal and human species. Data from the EID2 database [20].

From the EID2 database, we obtained records describing rickettsial species screened from the following carriers: arthropods (Figure 2A), domestic animals, wild mammals and humans (Figure 2B).

The number of rickettsial species recorded in arthropod species was highest in *Rhipicephalus sanguineus* (13 rickettsial species), followed by *Haemaphysalis longicornis* (7 species), *Ixodes ricinus* (6 species), *I. persulcatus* and *Amblyomma americanum* (5 species). All other arthropod species were recorded as harboring four or less rickettsial species (Figure 2A).

The highest number of rickettsial species was recorded in humans with 21 species, followed by the dog (*Canis lupus familiaris* with 15 species), the wolf (*Canis lupus* with 10 species), the cow (*Bos taurus* with 8 species), the cat (*Felis catus* with 7 species), the sheep (*Ovis aries* with 6 species), the goat (*Capra hircus* with 6 species), the house mouse (*Mus musculus* with 5 species) and the horse (*Equus caballus* with 5 species) (Figure 2B).

**Figure 3:**
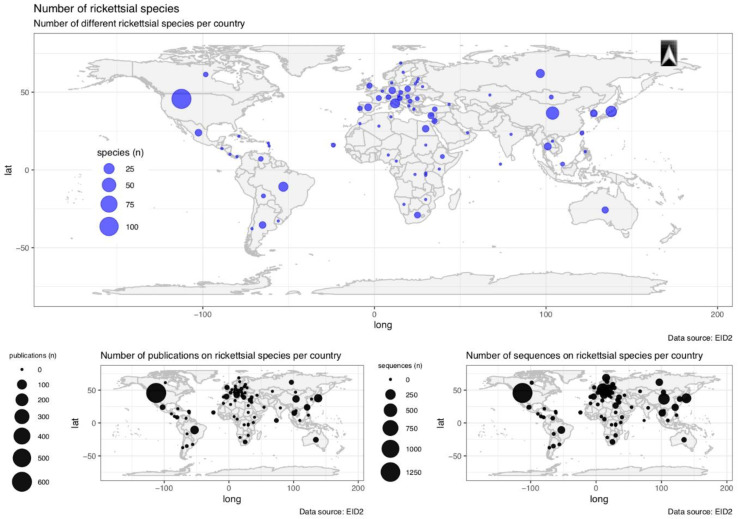
Geographical distribution of the richness of rickettsial species in the EDI2 database [20]. The hotspots of rickettsial species richness reflect the sampling effort as estimated by the number of publications (bottom left) or the number of sequences (bottom right) in the EDI2 database.

The geographical distribution of rickettsial species according to data extracted from EID2 showed a highly biased pattern, with high species richness in the US, Europe, China and Japan and low species richness in the tropical regions with the exception of Thailand (Figure 3). The observed pattern of rickettsial species richness is likely explained by the bias in screening effort, which is reflected in both the number of publications and the number of DNA sequences deposited in the NCBI nucleotide database (Figure 3), with the greatest numbers again recorded for US, Europe, China and Japan (along with the notable exception of Thailand).

The low screening effort of rickettsial species in South America, Africa and Southeast Asia may explain the low number of carrier species from these regions in the EID2 database (Figure 2).

### 3.4. Network Analysis of Rickettsial Species Among Reservoirs, Vectors and Humans

#### 3.4.1. Carrier Modularity

**Figure 4:**
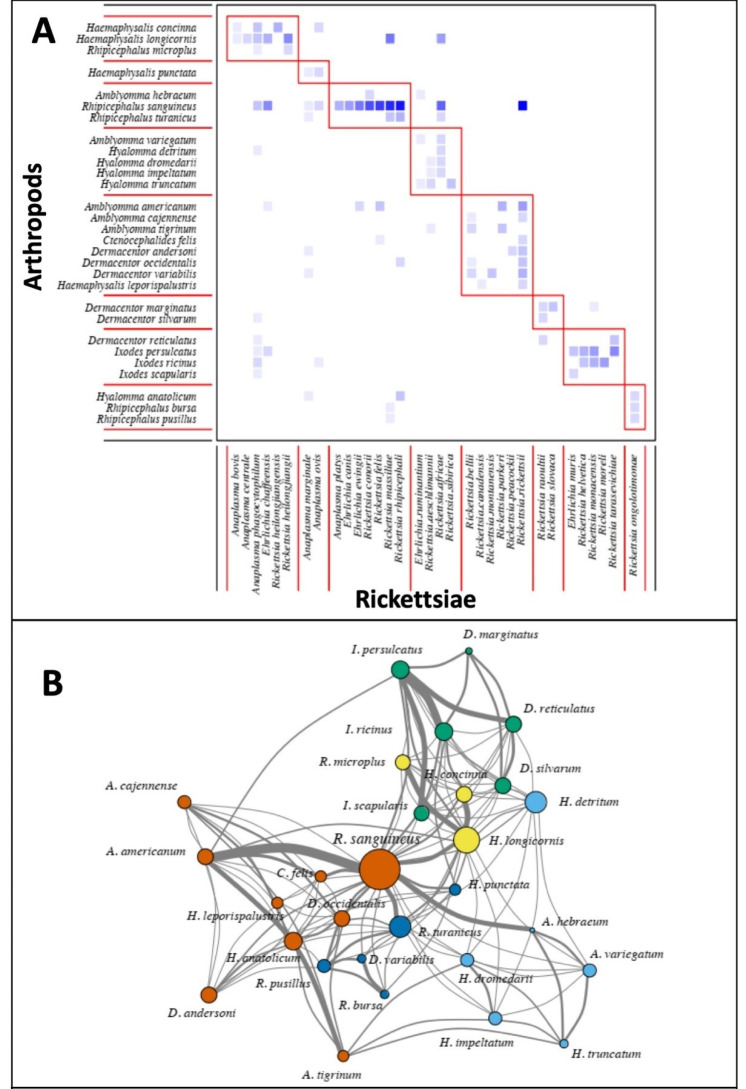
Composite panels of modules identified using (**A**) bipartite networks and (**B**) subgroups identified by unipartite networks subgroups (differentiated by colors) of shared rickettsial species among arthropod species The links among nodes (node = species) of the unipartite network depict shared pathogens/arthropod species (vertices were placed according to the Fruchterman–Reingold algorithm) with thickness of links proportional to number of rickettsial species shared and size of vertices proportional to the degree centrality of carriers (nodes). List of arthropod species is given in Figure 2. Data were extracted from the EID2 database [20].

**Figure 5:**
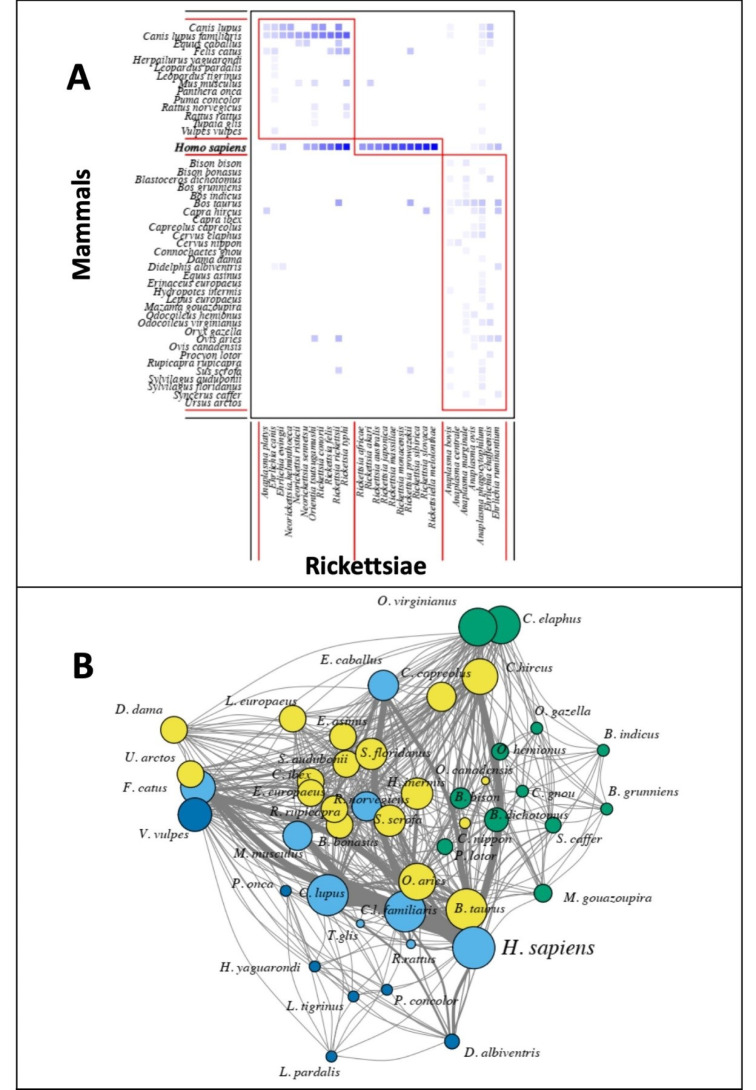
Composite panels of modules identified using (**A**) bipartite network and (**B**) subgroups identified by unipartite network subgroups (differentiated by colors) of shared rickettsial species among mammal species and humans. The links among nodes (node = species) of the unipartite network depict shared rickettsial species/mammals and humans (vertices were placed according to the Fruchterman–Reingold algorithm) with thickness of links proportional to number of rickettsial species shared and size of vertices proportional to the degree centrality of carriers (nodes). List of mammal species is given in Figure 2. Data were extracted from the EID2 database [20].

Using the presence information linking each carrier species with their rickettsial species, we obtained bipartite networks and unipartite projections in which each node was a carrier species, either arthropod or mammal species. Modules (bipartite)/subgroups (unipartite) were identified for all bipartite and unipartite networks of (i) shared rickettsial species among arthropod species (Figure 4) and (ii) shared rickettsial species among mammal species (Figure 5). Different numbers of modules were identified when using unipartite compared to bipartite networks.

In the case of arthropod carriers, the bipartite network identified eight modules (Figure 4A), such as the one grouping the carriers *Rhipicephalus turanicus*, *R. sanguineus* and *Amblyomma hebraeum* with the following rickettsial species: *Anaplasma platys*, *E. canis*, *E. ewingii*, *R. conorii*, *R. felis*, *R. massiliae* and *R. rhipicephali*. Unipartite network identified five modules of arthropod carriers. One module grouped *R. sanguineus*, *Amblyomma americanum*, *Dermacentor occidentalis*, *D. andersoni* and *Ctenocephalides felis*, among others; a second module grouped *H. longicornis*, *R. microplus* and *H. concinna*; the *Ixodes* species were grouped together with *D. reticulatus*, *D. marginatus* and *D. silvarum;* while other modules comprised species with less importance in the unipartite network, i.e., low degree centrality (Figure 4B).

Using the bipartite network, only three modules were identified for mammal carriers (Figure 5A), such as the one that groups humans (*H. sapiens*) with ten rickettsial species: *Rickettsia africae*, *R. akari*, *R. australis*, *R. japonica*, *R. massiliae*, *R. monacensis*, *R. prowazekii*, *R. sibirica* and *R. slovaca*. Four modules were identified using the unipartite network. The first module grouped *H. sapiens* with the dog, the wolf, the cat, the house mouse, the black rat and the tree shrew (*Tupaia glis*); the second one grouped the cow (*B. taurus*), the goat, the sheep, the pig, the rabbit and several wild cervids and small mammals; the third one grouped only wild mammals with the red fox, several wild cat species and the opossum (*D. albiventris*); the last module grouped several wild antelopes with the zebu (*Bos indicus*) and the domestic yak (*B. grunniens*) (Figure 5B).

The number of modules in unipartite or bipartite networks was always higher for arthropod carriers than for mammal carriers.

#### 3.4.2. Carrier Centrality

**Figure 6:**
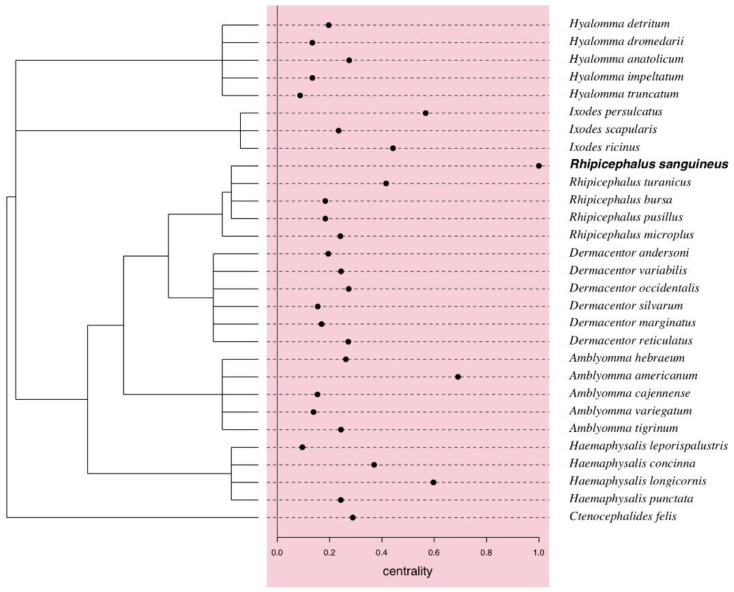
Centrality values of arthropod carriers of rickettsial species extracted from unipartite network (Figure 4A), with phylogenetic tree of arthropods obtained using ‘rotl’ [30] retrieved from the Tree of Life [31]. No significant phylogenetic signal for the centrality values was detected.

Central carriers are those contributing the most to the sharing of rickettsial species with other less central carriers. For arthropod species, these were *R. sanguineus* (degree centrality = 1) and *A. americanum* (degree centrality = 0.69), followed by species with decreasing importance in the degree centrality, i.e., architecture of the network: *Haemaphysalis longicornis* (0.60), *Ixodes persulcatus* (0.57), *I. ricinus* (0.44) (Figure 6).

**Figure 7:**
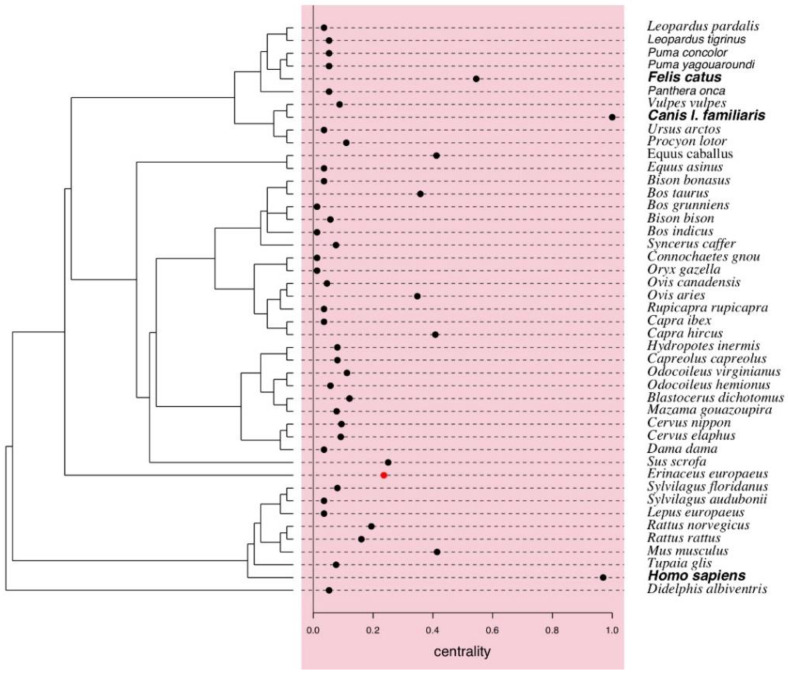
Centrality values of mammal carriers of rickettsial species extracted from unipartite network (Figure 4A), with phylogenetic tree obtained using ‘rotl’ [30] retrieved from the Tree of Life [31]. A significant phylogenetic signal for the centrality value of the European hedgehog was detected (red dot).

For mammal species, the dog showed the highest centrality (1.0), followed by the human species (0.97), the wolf (0.77), the domestic cat (0.54), the domestic mouse (0.41), the horse (0.41), the goat (0.41), the cow (0.36) and the sheep (0.35) (Figure 7). These domestic species were likely to share rickettsial species and acted as important hubs or connectors linking other carriers, particularly the wild mammals (Figure 6).

#### 3.4.3. Influence of Carriers’ Phylogeny on the Structure of Unipartite Networks

We found no significant phylogenetic signal on the centrality values of arthropod carriers, suggesting that closely related tick species were not closely related in the unipartite networks of shared rickettsial agents (Figure 6).

No global significant phylogenetic signal on the centrality values of mammal carriers was detected, with the exception of the European hedgehog (*Erinaceus europaeus*) (Figure 7).

## 4. Discussion

### 4.1. New Approaches for the Study of Rickettsial Diseases

The epidemiology of rickettsial zoonoses is mostly investigated by disease or by group of rickettsial species supposed to share similar epidemiological features. Published reviews mostly listed rickettsial species with their vectors, reservoirs and geographical distribution but could hardly capture a whole epidemiological ecology. We showed here that the application of data science, as a first approach used in disease ecology, may give useful tools to describe the global pattern of the epidemiology of rickettsial species.

First, we confirmed previous studies that emphasized the increasing incidence in rickettsial diseases at the onset of 1970 [13]. Using the GIDEON database and the records on outbreaks of rickettsial diseases, it was even possible to date the start of this increase of global outbreaks from 1971 (Figure 1).

Second, network analysis of association between rickettsial species and their carriers (vectors or reservoirs) extracted from the EID2 database appears to be a useful statistical tool. Investigation of the composition of modules bipartite networks or subgroups of unipartite networks of shared rickettsial species not only highlighted the importance of several carriers (vectors or reservoirs) but also exhibited their connections in the whole network of shared rickettsial species. Some arthropod species were identified in most of the bipartite modules and unipartite subgroups and noted for their centrality values in the unipartite network. These species were *R. turanicus*, *R. sanguineus*, *A. hebraeum, A. americanum, H. longicornis, H. concinna I. persulcatus, I. ricinus* and *C. felis*. These arthropod species harbor a significant number of shared rickettsial species and play a key role not only as vectors but also as bridges exposing various domestic animals and humans to various rickettsial diseases.

Four modules were identified using the unipartite network of shared rickettsial species among mammals. A first one grouped the human species with several domestic and commensal species such as the dog, the cat, the house mouse and the black rat, along with one peridomestic species, the tree shrew; a second module grouped other domestic species, the cow, the goat, the sheep, the pig, and the rabbit, with several wild cervids and small mammals; the third one grouped only wild mammals with the red fox and several wild cats but with the often peridomestic opossum; the last module grouped several wild antelopes with the domestic zebu and the domestic yak.

Hence, the unipartite network analysis confirmed the importance of domestic animals in sharing infectious diseases with humans and other wild animals [26,36]. Our results showed that the domestic species were likely to share rickettsial species with humans and to act as important hubs or connectors with other wild mammals. Interestingly, the number of modules, in both unipartite and bipartite networks, was always higher for arthropod carriers than for mammal carriers. This suggests that arthropod carriers have a greater impact on the architecture of the networks than the mammal carriers, which highlights the key role of arthropods in rickettsial transmission.

### 4.2. Factors of Emergence

#### 4.2.1. Vector and Reservoirs Carriers

The review of Swei et al. [19] synthesized the existing literature of emerging vector-borne zoonotic diseases and showed that a great number of 131 emerging vector-borne diseases from the years 1940 to 2018 were rickettsial diseases. Ixodidae ticks (*Ixodes, Dermacentor, Amblyomma spp.*) were recorded to transmit 37 (40%) emerging vector-borne diseases, which are mainly caused by Rickettsiaceae bacteria. The authors also found that the highest number of vector-borne diseases emerged in North America (27%) followed by Europe (21%) and Asia (20%). A similar geographical pattern was found using the EID2 database (Figure 2). Interestingly, the authors recorded the most commonly cited drivers for emergence in each reviewed reference and found that land use change was the first invoked factor for 26%, followed by international trade and commerce for 11%, while climate and weather-related factors accounted for 10%.

#### 4.2.2. Domestic and Commensal Mammal Carriers

Wild rodents and domestic animals such as dogs, cats and sheep are known as important hosts of spotted fever group rickettsial infection in humans [6]. In South America, opossum in the peridomiciliary area together with a high proportion of seropositive domestic animals in households, such as the horse, the donkey or the domestic dog, were associated with rickettsial seropositivity in humans [37]. Our results showed the importance of these domestic animals (dog, cat and horse) as well as commensal and peridomestic animals (e.g., opossum) in the sharing of rickettsial species using network analysis. The likely explanation is the close relationship of domestic and commensal animals associated with human activities or human settlement that favors transmission of zoonotic diseases. This has been already observed using network analysis for the sharing of diseases among domestic animals and humans [26] or the sharing of viruses among wildlife, domestics and humans [36].

#### 4.2.3. Climate Change

Although the reviews of Jones et al. [18] and Swei et al. [19] questioned the importance of climate factors and climate changes in the epidemiology of rickettsial diseases, Parola et al. [38] provided evidence of a warming-mediated increase in the aggressiveness of the tick *R. sanguineus*, leading to an increase of human attacks, associated with clusters of cases of spotted fever caused by *R. conorii* and *R. massiliae* in France and Italy in 2007.

Using the EID2 database, McIntyre et al. [39] investigated the climate sensitivity of important human and domestic animal pathogens in Europe. The pathogens were selected using a prioritization method based on the H-index of the diseases [40]. Unfortunately, among the 3628 pathogen species, only 157 were selected, with only one rickettsial species, *Anaplasma phagocytophilum*. While a great majority of the 157 studied pathogens showed no climate drivers or only one climate driver that can affect their epidemiology, *A. phagocytophilum* was one of the few pathogens showing a high number of influential climate drivers. The list of the climate factors comprised moisture, rainfall, temperature, altitude, climate change and vegetation (a likely habitat driver). All of these climate and habitat drivers could potentially affect the tick vector as well as many reservoir species (e.g., rodents, other small mammals and deer) of *A. phagocytophilum*.

Climate change has not been investigated in detail in this context, except for in the case of scrub typhus, a rickettsial disease caused by *Orientia tsutsugamushi* and transmitted to humans through infected chigger mites [41]. One million cases of scrub typhus occur every year, while one billion persons are considered at risk [15]. The resurgence of scrub typhus has been reported in several countries of the “*tsutsugamushi* triangle” [14,42,43,44,45]. Li et al. [46] estimated the effects of diverse climate variables on the incidence of scrub typhus in the city of Guangzhou from 2006–2012. Controlling for several potential confounding factors, they showed that each 1 °C rise in temperature corresponded to an increase of 15% in scrub typhus cases by month.

#### 4.2.4. Land Use Change

Murray et al. [47] characterized the epidemiology of typhus group rickettsiosis in Texas (USA) from 2003 to 2013, showing a geographic expansion of the number of diagnosed cases over time. However, the study did not investigate the effects of any factor explaining the northern shift of the incidence, which could be in relation to a change in abundance of the flea vectors or the animal reservoirs in relation to habitat change.

The recent meta-analysis of Shah et al. [48] is the more comprehensive study on the effect of agricultural land use changes on the risks of infectious diseases in Southeast Asia. Among 77 studies, 13 studies concerned rickettsial diseases, with five for scrub typhus, four for murine typhus (*R. typhi*), two for spotted fever group, one for *R. felis* flea-borne spotted fever and one for *R. conorii* spotted fever. Typhus was associated with non-specific agricultural changes, whereas *R. conorii* and other rickettsial diseases of spotted fever group showed significant association with livestock farming. Interestingly, the two flea-borne diseases (*R. typhi* and *R. felis*) showed no association with agricultural land use changes. A lack of association is explained by the observation that murine typhus is mostly associated with the urban environment.

### 4.3. Implications for Public Health

Disease ecology is an integrative science, integrating environment, ecology and evolution of diseases and taking into account the scaling effect from local to global scales. Disease ecology is also a collaborative science that aims to involve biologists, clinicians, and public and animal health practitioners. As emphasized in the present study, disease ecology is highly dependent on data, which should be high-quality (requiring quality control), well-described (adding metadata), geo-referenced, and accessible (open data) following ethical standards [49]. Disease ecology allows the epidemiologist and the health practitioner to capture the dynamics of disease transmission in a more integrative/holistic approach. Although the first aim of disease ecology is to describe transmission patterns and likely transmission mechanisms, a second aim is to develop scenarios of disease transmission, for which some recent progress has been made.

### 4.4. Current Limitations

A first limitation is that many of the Rickettsiales listed in EID2 database have no experimental or epidemiological or clinical/veterinary support for their pathogenicity. A second limitation is that our approach necessitates continuously updating databases (EID2, GIDEON, etc.), taking into account changes in taxonomy (valid species, synonymy, etc.); such databases are not updated in real time. For example, and only for Southeast Asia, Low et al. [5] summarized the newly discovered regional rickettsial species, including *Rickettsia thailandii*, *Candidatus R. sepangensis*, *Candidatus R. johorensis*, *Candidatus R. laoensis*, *C. Rickettsia mahosotii*, *C. Rickettsia khammouanensis* and *C. Anaplasma pangolinii*. The last limitation is the lack of associated ecological data that would help to contextualize the patterns depicted by the network analyses. Nevertheless, although updating databases and data records of rickettsial species is necessary, it will not change the methodological approach proposed here to investigate the disease ecology of rickettsial species using the tools of data science.

## Figures and Tables

**Table 1:** Emerging rickettsioses (from the supplementary materials of Jones et al. [18] and Swei et al. [19]) from 1920 to 2016 (with the exception of novel rickettsial characterization).

Year	Rickettsial Agents	Country	Likely Causes of Emergence
1920	*Rickettsia conorii*	Europe, Africa	Climate and weather
1930s	*R. sibrica*	Asia	? (Unspecified)
1946	*R. akari*	US	Human demographics and behavior
1946	*R. australis*	Australia	? (Unspecified)
1948	*Orientia tsutsugamushi*	Japan	War and famine
1983	*R. typhi*	US	International travel and commerce
1984	*R. japonica*	Japan	? (Unspecified)
1986	*Ehrlichia canis*	US	Land use changes
1990	*Anaplasma phagocytophilum*	US	Land use changes
1990	*E. chaffeensis*	US	Land use changes
1990	*R. honei*	Thailand	International travel and commerce
1991	*R. felis*	US	?
1992	*R. africae*	Zimbabwe	International travel and commerce
1995	*R. prowazekii*	Burundi	War and famine
1996	*R. mongolotimonae*	France	International travel and commerce
1996	*R. slovaca*	France	Land use changes
1997	*R. helvetica*	Sweden	Land use changes
2002	*R. aeschlimannii*	Africa	Agricultural industry changes
2005	*R. monacensis*	Europe	? (Unspecified)
2006	*R. kellyi*	India	? (Unspecified)
2006	*R. massiliae*	South America, Europe	Climate and weather
2007	*Candidatus Neoehrlichia spp.*	Europe	Human susceptibility to infection
2008	*R. philipii*	US	Human demographics and behavior
2009	*R. conorii subsp. caspia*	Europe	Land use changes
2011	*E. muris-like agent*	US	Human susceptibility to infection
2011	*R. tamurae*	Japan, Laos	? (Unspecified)
2012	*R. montanensis*	US	? (Unspecified)
2012	*R. tarasevichiae*	China, Russia	? (Unspecified)
2016	*R. indica*	Japan	International travel and commerce

## Supplementary Materials

The following are available online at https://www.mdpi.com/2414-6366/5/2/64/s1, Table S1: EID2 database.
